# Esketamine-induced dissociation: just a side effects or a potential antidepressant effect booster?

**DOI:** 10.1192/j.eurpsy.2025.1359

**Published:** 2025-08-26

**Authors:** F. Raffone, P. Sarasso, M. Billeci, G. Di Petta, V. Martiadis

**Affiliations:** 1 Department of Mental Health, Asl Napoli 1 Centro, Naples; 2 Department of Psychology, University of Turin, Turin; 3 Department of Mental Health, Asl Napoli 2 Nord, Pozzuoli; 4 Department of Mental Health, Asl Napoli 2 Nord, ozzuoli; 5 Department of Mental Health, Asl Napoli 1 Centro, Neaples, Italy

## Abstract

**Introduction:**

Dissociative experiences are considered typical esketamione’s side effects. Several investigations have have shown that ketamine/esketamine-induced dissociation may be linked to improved responsiveness, although other studies have not supported this association. Despite these controversial findings, it’s possible that dissociative experiences are crucial to the antidepressant effects, at least in specific subtypes of depression. In fact, current understanding of the therapeutic potential of ketamine (the anaesthetic from which esketamine is derived) converges on the so-called “relaxed prior hypothesis”, suggesting that glutamatergic blockade up-weights bottom-up surprising somatosensory/affective states. Consequently, ketamine improves short-term plasticity in depression by enhancing sensitivity to interoceptive signals.

**Objectives:**

This study describes and discusses two case studies in which the experience of esketamine dissociation was particularly effective in enhancing antidepressant effects.

**Methods:**

We selected 2 case studies for their paradigmatic description of “depersonalised depression” (Entfremdungsdepression). Patients were interviewed both 4 weeks before and at the peak of esketamine effects during a 6-month treatment. Following a neurophenomenological approach, we combined subjective reports from unstructured clinical interviews with review of previous objective neuroimaging results and neurocomputational models to elucidate the relationship between esketamine’s effects and interoceptive sensitivity. Patients were administered the HAM – D at T0 and T6 and the Dissociative Experience Scale (DES-II) and the Structured Clinical Interview for Depersonalization-Derealization Spectrum (SCI-DER) at T0.

**Results:**

According to our observations, esketamine-induced dissociation may be particularly effective in the depersonalised depression subtype, in which interoceptive awareness and interaffectivity are significantly impaired. In particular, disembodiment may suspend previously acquired patterns of feeling, perception and behaviour. As previously described by Richard Yansen, ketamine disrupts the assertion of bodily tensions so that they cannot tighten in the way they used to tighten, and they cannot hold back emotionally in the way they used to hold back: then many of the feelings behind the armour can pour out with a softening, cathartic release. Our patient D.P. sketches this phenomenon nicely when he says that “esketamine makes you change your own personal attitude, your own grammar”.

**Conclusions:**

Consistent with previous findings, we suggest that esketamine-induced disembodiment allows for a temporary window of psychological plasticity and heightened sensitivity with the body regaining its permeability to affective affordances. Future research should address the relationship between esketamine-induced disembodiment, sensitivity to interoceptive signals, and depression outcomes.

**Disclosure of Interest:**

None Declared

